# Immobilization of silver nanoparticles and silver iodide within bamboo fabrics for wastewater treatment

**DOI:** 10.1038/s41598-025-93188-x

**Published:** 2025-04-01

**Authors:** Mohamed Rehan, El-Amir M. Emam, Hossam E. Emam

**Affiliations:** 1 Department of Pretreatment and Finishing of Cellulosic Based Textiles, Textile Research and Technology Institute, National Research Centre, 33 EL Buhouth St., 12622 Dokki, Giza, Egypt; 2 Faculty of Applied Arts, Textile Printing, Dyeing and Finishing Department, Helwan University, 11795 Cairo, Egypt

**Keywords:** Bamboo fabrics, AgI, Ag NPs, Water treatment, Antimicrobial, Catalytic, Photocatalytic, Environmental sciences, Natural hazards, Chemistry, Materials science

## Abstract

Pathogenic microorganisms and dyes are the main sources of water pollution. These pollutants are extremely hazardous and may harm aquatic life and human health. As a result, removing these pollutants is critical in assessing contamination risks and mitigating potential health hazards. To effectively remove pathogenic microorganisms and dyes from wastewater, an efficient multi-functional material was designed based on AgI, Ag NPs, and Ag NPs@AgI immobilized on bamboo fabrics as a support substrate. The water disinfection aptitude of the modified bamboo fabrics was evaluated against different microorganisms. The results showed that the Ag NPs@AgI@bamboo showed excellent antibacterial activity against *S. aureus* (88%) and *E. coli* (90%) as well as perfect antifungal activity against *C. albicans* (82%). Methylene blue (MB) was used as a pollutant model to test the catalytic and photocatalytic activity of modified bamboo fabrics. The results show that Ag NPs@AgI@bamboo was highly efficient in removing the MB dye via reduction (90%) after 60 min or photodegradation (93%) after 6 h of UV light irradiation. The pseudo-first-order kinetic study shows that Ag NPs@AgI@bamboo possessed outstanding catalytic reduction and photocatalytic degradation activities toward MB.

**Supplementary Information:**

The online version contains supplementary material available at 10.1038/s41598-025-93188-x.

## Introduction

Water pollution has become a worldwide environmental problem due to population growth, industrialization, and increased waste discharge, especially in developing nations. The absence of comparable water resources exacerbates such a risky problem^1^. Industrial and agricultural waste are the main reasons for water pollution^2^. Pathogenic microorganisms, heavy metals, and organic pollutants are the most risky categories of water pollutants^3–5^. One of the biggest risks to human health has been determined to be microbial contamination. Numerous microorganisms from various groups, such as bacteria, viruses, and protozoa, can be found in water. Different microbial communities in wastewater treatment facilities are caused by other sources of microorganisms in wastewater. The greatest microbiological risks are associated with drinking water contaminated by human or animal waste^6,7^.

Organic dyes are commonly found in industrial wastewater, seriously contaminating the environment. Dyes are used by a variety of industries, including paper, leather, cosmetics, textiles, and apparel, to add color to their products. Every year, roughly 7 × 10^5^ tons of synthetic dyes are used worldwide. Because dyes contain many aromatic rings that are hardly decomposed via traditional methods, they are mainly ascribed to be non-biocompatible. Furthermore, some of these dyes poison marine biological activity. Water dyes can strongly absorb most sunlight, which lowers the dissolved oxygen content and inhibits the growth of aquatic life^8–10^.

Wastewater is treated using a variety of techniques, including reverse osmosis, coagulation-flocculation, ion exchange, solvent extraction, chemical precipitation, and ultrafiltration. Despite their efficiency, these methods have several disadvantages, including high energy consumption, high maintenance and operating costs, and a significant amount of sludge that requires proper disposal. These restrictions have led to the emergence of creative substitute technologies that employ economical, ecologically conscious, and cost-effective methods^11^.

The manufacturing of functional materials was widely investigated by researchers as a possible remedy for the environmental problem of water pollution^12–22^. The most recent and popular methods for treating wastewater are adsorption^23–26^ and photo-catalysis^27–29^ because they are easy to use, costless, and environmentally benign^30,31^. As a potential strategy to increase efficacy, nanotechnology has been used to synthesize the majority of functional materials used in water filtration to date^32–34^. In recent years, photo-catalysis has gained popularity as a means of treating a variety of pollutants, such as dyes^35–37^, pharmaceuticals^38–40^, and pathogenic microorganisms^41,42^. The primary goal of the research is to create stable, non-toxic, commercially viable, and photo-corrosion-resistant catalysts. Heterogeneous photo-catalysis, which uses photo-chemically active semiconductors as a photo-catalyst, has garnered a lot of attention in recent decades due to its enormous potential for converting photon energy into chemical energy and breaking down hazardous pollutants^43^. Reactive oxygen species (ROS) production is a hallmark of heterogeneous photocatalysis^44,45^. As photo-catalysts, various semiconducting materials have been used, including oxides (TiO_2_^46,47^, ZnO^48,49^, CeO_2_^50^, ZrO_2_^51^, and WO_3_^31^) and sulfides (CdS^52^, and ZnS^53^).

Because of its remarkable performance in photocatalytic processes, such as high absorption coefficients in a broad UV/Vis/NIR spectral range and enhanced resistance to degradation due to the Plasmonic particles’ strong surface, Plasmonic photo-catalysis (silver halide/silver nanoparticles AgX/Ag) is one of the most promising techniques^20,54–59^. AgX, X = Cl, Br, I is a key photosensitive material in photography. AgX is a new type of photocatalytic active material that is activated by light irradiation. AgX can absorb photons under light irradiation and produce electron-hole pairs. However, AgX will experience undesired, uncontrollable photodecomposition that reduces its activity in practical applications when photoinduced electrons from its photodecomposition combine with interstitial Ag^+^ ions to form an Ag^0^ cluster^60,61^.

A new class of Plasmonic photo-catalysts, silver/silver halide nanoparticles (Ag@AgX) is reported to be highly applicable because of their high stability, nontoxicity, resistance to degradation, and visible response. Because of the strong surface Plasmon resonance (SPR) effect of silver nanoparticles (Ag NPs), the light absorption ranges of Ag/AgX shifted from the ultraviolet to the visible region. Increased electron and hole inhibition in the composite can also lead to high visible light catalytic activity. Ag@AgX nanoparticles have exceptional antimicrobial properties as well^59,60,62^. AgI stands out among AgX because of its narrow band gap (2.80 eV), high ionic conductivity, and capacity to boost TiO2’s sensitivity to visible light. Furthermore, a wide range of microorganisms are thought to be effectively inhibited by AgI. Remarkably, the Ag@AgI system demonstrated excellent stability and potent photocatalytic activity under light^63–70^.

One support substrate that shows promise in addressing the aforementioned issues is cellulosic fibers. The enrichment with surface hydroxyl groups (-OH) found in cellulosic fibers makes them suitable as a substrate for immobilizing AgX@Ag. Strong electrostatic interaction is anticipated to be the mechanism by which the nanoparticles bind to the cellulosic fibers. Bamboo fiber is a renewable cellulosic fiber that is produced by the bamboo plant. It’s a great fiber that is biodegradable and safe for the environment.

To the best of our knowledge, no reports have been published on the modification of bamboo fabrics with Ag NPs, AgI, or Ag NPs@AgI and their use in environmental applications. AgI@bamboo, Ag NPs@bamboo, and Ag NPs@AgI@bamboo photocatalysts are prepared herein easily and inexpensively under the effect of infrared irradiation. The obtained AgI@bamboo, Ag NPs@bamboo, and Ag NPs@AgI@bamboo are applied in the degradation of methylene blue dye.

## Experimental

### Materials and chemicals

Bamboo fabrics (single jersey bamboo 100% white 30/1, 200 g/m^2^) were obtained from Hensi Company, Egypt. Bamboo fabrics were washed for 30 min in hot water (70 °C), and then rinsed with deionized water at room temperature. Silver nitrate (AgNO_3_, 99.5%, Panreac, Barcelona − Spain). Potassium iodide (NaI, ≥ 99%), sodium borohydride (NaBH_4_, ≥ 98%,), and methylene blue (MB) were obtained from Sigma-Aldrich-Germany. Sodium hydroxide (NaOH, 99%, from S.D. Fine Chemical Limited, Mumbai – India). All the chemicals and reagents were used without any further purification.

### Synthesis of AgI@bamboo

AgI was in situ deposited onto the bamboo fabrics under the effect of infrared irradiation via one-pot two-steps. 5 g of bamboo fabric was immersed in an aqueous silver nitrate solution (100 ppm) with a liquor ratio of 1/5 at room temperature for 10 min. The mixtures were transferred to the hydrothermal autoclave reactor and placed in the infrared machine “Infra-Red Dyeing Machine,” pre-adjusted at 80 °C for 30 min. Under infrared irradiation, Ag+ ions adsorb and diffuse into the bamboo fabric surface via electrostatic interaction with hydroxyl groups and carboxyl groups on the bamboo fabric surface. The bamboo fabrics were washed with deionized water and then dipped into a 100 ppm aqueous solution of KI (100 ppm) with a liquor ratio of 1/5 at room temperature for 10 min. The mixtures were transferred to the infrared machine at 80 °C for 30 min. Under infrared irradiation, the I⁻ ions bind with Ag⁺ absorbed on the bamboo fabrics, forming AgI incorporated into the bamboo fabrics. The AgI@bamboo was rinsed with running deionized water and dried at room temperature.

### Synthesis of ag NPs@bamboo and ag NPs@AgI@bamboo

5 g of bamboo fabrics and AgI@bamboo were stepwise immersed in 250 ml of 0.1 N NaOH and stirred for 10 min at room temperature. Silver nitrate solution (100 ppm) was added dropwise to the container with stirring, and then the temperature was raised to 70 ± 3 °C. After 45 min, the fabrics were taken out, squeezed, and rinsed with tap water for neutralization and then squeezed again. AgI@bamboo, Ag NPs@bamboo, and Ag NPs@AgI@bamboo were dried at 75 ± 5 °C before analysis and characterization.

### Characterization of bamboo fabrics

The morphologies of the bamboo fabric samples were investigated using a scanning electron microscope, (SEM) (ZEISS, LEO 1530 Gemini) with an acceleration voltage of up to 30 kV equipped with a LaB6 electron gun and a Philips-EDAX/DX4 energy-dispersive spectroscope (EDS). X-ray diffraction (XRD) was used for phase identification and crystal structural analysis for bamboo fabric samples (PANalytical X’pert PRO PW 3040/60 (Netherlands) X-ray diffraction fitted with a Cu Kα (λ = 0.154 nm) radiation source in range 2θ = (10°–80°). The infrared spectrum for the bamboo fabric samples was measured by using a Spectrum 65 FTIR spectrometer (PerkinElmer Co., Ltd., MA, and USA). The spectra were in the range of 4000 cm^− 1^ to 500 cm^− 1^. The X-ray photoelectron spectroscopy (XPS, X-Ray – FG ON, 400 μm) was used to perform the XPS spectral analyses for the modified bamboo fabrics. Samples were exposed to low energy electrons with a voltage of 0.1 eV, using the hybrid mode of electrostatic/magnetic lenses and 22 scans, while monochromatized α-irradiation was used for the photoelectrons excitation. The colorimetric analysis including K/S, CIE LAB color space data, and absorbance of the bamboo fabrics samples were measured through a spectrophotometer with pulsed xenon lamps as a light source (Ultra Scan Pro, Hunter Lab, USA.

### Evaluation of antimicrobial activities

The antimicrobial activities of the tested bamboo fabric samples were examined against some targeted pathogenic microorganisms obtained from the American-type culture collection (ATCC; Rockville, MD, USA). The tested organisms were *Staphylococcus aureus* ATCC- 47,077 (*S. aureus*), *Escherichia coli* ATCC-25,922 (*E. coli*), and *Candida albicans* ATCC-10,231 (*C. albicans*), with cell numbers of 3 × 10^6^, 4 × 10^6^ and 3 × 10^6^ cfu/mL, respectively. The stock cultures of pathogens used in this study were maintained on nutrient agar slants at 4 °C. Seventy microliters of bacterial and yeast cells of each pathogen were added to 10 ml of nutrient broth medium. The sample discs were added to inoculated tubes. The tubes were incubated with shaking at 37 °C for 24 h (in the dark and under UV light) except the yeast strain that was incubated at 28 °C for 24 h (in the dark and under UV light) followed by the measurement of the optical density by spectrophotometer at 550 nm, and then the bacterial reduction is estimated.

### Evaluation of catalytic properties

The catalytic activity of bamboo fabrics, AgI@bamboo, Ag NPs@bamboo, and Ag NPs@AgI@bamboo was analyzed for the degradation of the organic dye methylene blue (MB). The reaction mixture was mixed thoroughly, and NaBH_4_ was used as a reducing agent^38^. The catalytic performance was tested by dipping 0.1 g of bamboo fabrics, AgI@bamboo, Ag NPs@bamboo, and Ag NPs@AgI@bamboo in a 10 ml solution of MB (10 mg/L pH 6.5) with 1 ml of 0.05 M NaBH_4_ at 25 °C and constant stirring. For comparison, the process was carried out in the absence of NaBH_4_, which performed as an adsorption process. The catalytic process was tracked via UV–vis spectra within the range of 400 to 800 nm. The catalytic dye degradation was monitored at time intervals (5, 10, 15, 20, 30, 40, 50, and 60 min) by taking 2 ml of solution in a cuvette after regular intervals of time, and its absorption spectra were recorded with a UV-Vis spectrophotometer (Shimadzu UV 1800, Tokyo, Japan). The catalytic fraction of the dye can be expressed by Eq. (1). For further analysis of the reaction mechanism, pseudo-first-order kinetics and pseudo-second-order kinetics models were evaluated, and these two kinetic models are expressed by Eqs. (2) & (3).

1 $${\text{The}}\,{\text{catalytic}}\% = {\text{A}}_{0}^{ - } {\text{A}}_{{\text{t}}} /{\text{A}}_{0} \times 100$$

Where A_0_ is the absorbance of MB solution before the reaction and A_t_ is the absorbance of MB at time t of the catalytic reaction.

2 $${\text{Ln}}\left( {{\text{C}}/{\text{C}}^{0} } \right) = {\text{K}}_{1} {\text{t}}$$

3 $$1/{\text{C}}{-}1/{\text{C}}^{0} = {\text{K}}_{2} {\text{t}}$$

The initial and time t concentrations of the solution are represented by C^0^ and C, respectively. Degradation time is denoted by t (min), and rate constants of first and second order are denoted by K_1_ (min^− 1^) and K_2_ (L mg^− 1^·min^− 1^).

### Evaluation of photocatalytic properties

The photocatalytic activity of bamboo fabrics, AgI@bamboo, Ag NPs@bamboo, and Ag NPs@AgI@bamboo was evaluated by photocatalytic degradation of methylene blue (MB) in water under UV light irradiation in UV (320–400 nm) was performed with a laboratory-constructed illumination box equipped with one F15W/T8 black light lamp (315 nm < λ < 380 nm and photon energy 2.26–3.94 eV emitting 668 µW cm^− 2^ at a distance of 5 cm). Tests were performed by following the change in maximum absorbance of MB at 664 nm during the time of irradiation. 1 g of bamboo fabrics, AgI@bamboo, Ag NPs@bamboo, and Ag NPs@AgI@bamboo immobilized in 50 ml MB (10 mg/L pH 6.5) was magnetically stirred in the dark for 30 min to establish an adsorption-desorption equilibrium between the cotton fabric samples and MB under room air equilibrated conditions. Then it was irradiated for a certain period with constant stirring under UV light. After every given interval of irradiation time (1, 2, 3, 4, 5, 6 h), about 2 mL of the solution was collected and analyzed using a spectrophotometer^38^. For comparison, the process was performed in the dark, which is expressed for the adsorption process. The MB concentration was determined by changing the maximum absorption at a wavelength of 665 nm in UV absorption spectra versus irradiation time. The photocatalytic degradation was determined by the following Eq. (4):

4 $${\text{Photocatalytic}}\,{\text{degradation}}\% ~ = ~{\text{A}}_{0}^{ - } {\text{A}}_{{\text{t}}} /{\text{A}}_{0} . \times {\text{1}}00 = ~{\text{C}}_{0} ~ - ~{\text{C}}_{{\text{t}}} /{\text{C}}_{0} ~ \times 100$$

Where A_0_ is the initial absorbance and A_t_ the variable absorbance at various irradiation times. C_0_ represents the initial concentration of MB, C_t_ the concentration at various times of light irradiation,

### Statistical analysis

The measurements were performed in triplicate and the net averages were measured and expressed by Mean ± SD. The data was drawn with Microsoft Excel (version 2016).

## Results and discussion

### Proposed mechanism in situ synthesis of agI, ag NPs, and ag@AgI

In-situ synthesis of nanoparticles onto the bamboo fabric surfaces was preceded via infrared-assisted one-pot incorporation of AgI, Ag NPs, and Ag@AgI NPs. Bamboo fabric surfaces provide sites for nanoparticle nucleation due to the presence of many hydroxyl groups and carboxyl groups on their surfaces. The bamboo fabric was used as a solid support (i.e., template) for the deposition of the nanoparticles. Firstly, bamboo fabrics were impregnated in an aqueous silver nitrate solution. The electrostatic interaction between negatively charged hydroxyl (-OH) groups (major site) and/or carboxyl (-COOH) groups (minor site) on the bamboo fabric and the positive charge of Ag^+^ ions causes the Ag^+^ ions to adsorb and diffuse into the surface of the bamboo fabric^57^.

$${\text{Bamboo}}\,{\text{fabric}} - {\text{OH}} \to {\text{Bamboo}}\,{\text{fabric - O}}^{ - }$$

$${\text{Bamboo}}\,{\text{fabric}} - {\text{O}}^{ - } + {\text{Ag}}^{ + } \to {\text{Bamboo}}\,{\text{fabric - O - Ag}}^{ + }$$

$${\text{Bamboo}}\,{\text{fabric - COOH}} \to {\text{Bamboo}}\,{\text{fabric - COO}}^{ - }$$

$${\text{Bamboo}}\,{\text{fabric - COO}}^{ - } + {\text{Ag}}^{ + } \to {\text{Bamboo}}\,{\text{fabric - COO - Ag}}^{ + }$$

In the second step, the bamboo fabrics containing Ag^+^ were dipped into an aqueous solution of KI. When the bamboo fabrics are exposed to infrared irradiation, Ag^+^ absorbed by the bamboo fabrics transforms to AgI. There are three stages to the in-situ formation of Ag NPs integrated into bamboo fabrics and bamboo fabrics containing AgI: pre-nucleation, nucleation, and growth. Silver ions (Ag^+^) can be efficiently reduced into silver nanoparticles (Ag^0^) by bamboo fabrics, which were also stabilized through interaction with their -OH and -COOH groups^71^.

$${\text{Bamboo}}\,{\text{fabric - O - Ag}}^{ + } + {\text{K}}^{ + } {\text{I}}^{ - } \to {\text{Bamboo}}\,{\text{fabric - O - AgI}}$$

$${\text{Bamboo}}\,{\text{fabric - COO - Ag}}^{ + } + {\text{K}}^{ + } {\text{I}}^{ - } \to {\text{Bamboo}}\,{\text{fabric - COO - AgI}}$$

Bamboo and bamboo fabrics containing AgI function as a substrate for even distribution and fixation in this reaction, as well as a reducing agent and stabilizer of silver ions to stop the resulting silver nanocrystals from aggregating^72^. Immersion of bamboo fabrics and bamboo fabrics containing AgI in NaOH caused swelling of the fabrics, which aided in the distribution of silver ions (Ag^+^) inside the fabric matrix, increasing the homogeneity and mobility of the silver ions inside the matrix and enhancing the formation of Ag nuclei and controlling their growth^73^. During the swelling process, non-active fabrics are converted into alkali-activated fabrics (fabrics-COONa). Additionally, the alkali pretreatment increased the pH to around 12, which aids in the conversion of Ag^+^ to Ag^0^. In the pre-nucleation step, the functional groups of bamboo fabric molecules (-COO-Na and OH groups) form ion exchange interactions and/or complexation with silver ions (Ag^+^). As a result, the electrostatic drive generates an attractive force and a Van der Waals force, causing silver ions to be evenly and closely adsorbed and anchored on the surface of bamboo fabrics and bamboo fabrics containing AgI. In the nucleation step, by raising the temperature to 70 °C, silver ions (Ag^+^) are reduced to nano-sized (Ag^0^) inside the matrix by bamboo fabric end aldehyde groups. In the growth step, the silver atoms are reached supersaturation; they agglomerate to form nuclei and seed crystals. The formed seed played a major role in the formation of anisotropic structures. Finally, Ag NPs were grown, and with the aid of a fabric matrix, they were well-stabilized and protected, where, Ag metal cluster surfaces are likely to be anchored through the strong interaction between the Ag NPs surface and the oxygen atom of the functional groups (-COO- and -OH) of bamboo fabrics^74,75^.

### Characterization of bamboo fabrics

**Fig. 1:**
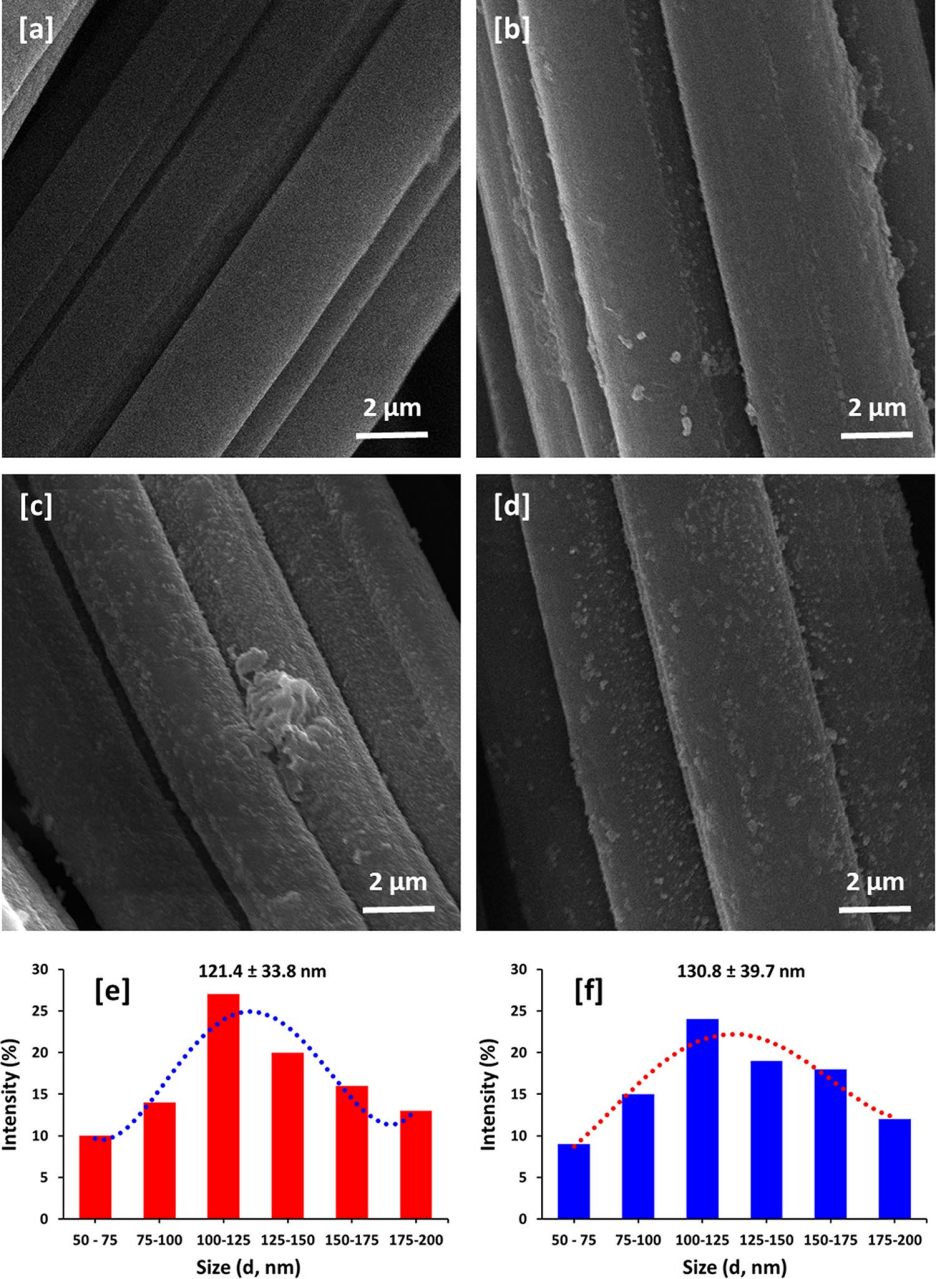
Scanning electron micrographs for the bamboo fabric samples; (**a**) bamboo blank, (**b**) AgI@bamboo, (**c**) Ag NPs@bamboo, and (**d**) Ag NPs@AgI@bamboo. Size distribution estimated from SEM; (**e**) Ag NPs@bamboo and (**f**) Ag NPs@AgI@bamboo.

The morphological and structural changes in the bamboo fabric as a result of the modification by the AgI, Ag NPs, and Ag NPs@AgI NPs were investigated by SEM, EDX, XRD, and FTIR analysis. The morphology, microstructure, and elemental composition details of bamboo fabrics, AgI@bamboo, Ag NPs@bamboo, and Ag NPs@AgI@bamboo were investigated by SEM-EDX. The SEM image of bamboo fabrics (Fig. 1a) shows that the fibers’ length is lined with fibrils, which make up the fabric’s morphology. These fibers are smooth, sleek, and have straight stripes. Figure 1b–d show the SEM images of AgI@bamboo, Ag NPs@bamboo, and Ag NPs@AgI@bamboo, respectively. The surface of the bamboo fabrics becomes rough after being modified with nanoparticles. SEM image of AgI@bamboo (Fig. 1b) demonstrates that the bamboo fabrics were covered with a uniform and low distribution of AgI nanoparticles. SEM image of Ag NPs@bamboo (Fig. 1c) shows that the bamboo fabrics were covered with some irregular spherical particles and disordered arrangements with agglomeration. SEM image of Ag NPs@AgI@bamboo (Fig. 1d) shows that the bamboo fabrics were covered with a continuous and heterogeneous layer of tiny particles. AgI@bamboo, Ag NPs@bamboo, and Ag NPs@AgI@bamboo SEM images demonstrate that the nanoparticles are small and seem to be well embedded (incrusted) within the network made up of bamboo nanofibrils.

The size distribution and average size were estimated from the presented micrographs by using 4 pi software, and the data is presented in Fig. 1. The average particle size was 121.4 nm in the case of Ag NPs@bamboo (Fig. 1e), while the average particle size was 130.8 nm in the case Ag NPs@AgI@bamboo (Fig. 1f). The Ag particles are see agglomerated in fabric, however, particle size is ranged in 50–200 nm. Therefore, it can be considered as nanoparticles as some particles are less than 100 nm and the all particles are less than 200 nm.

**Fig. 2:**
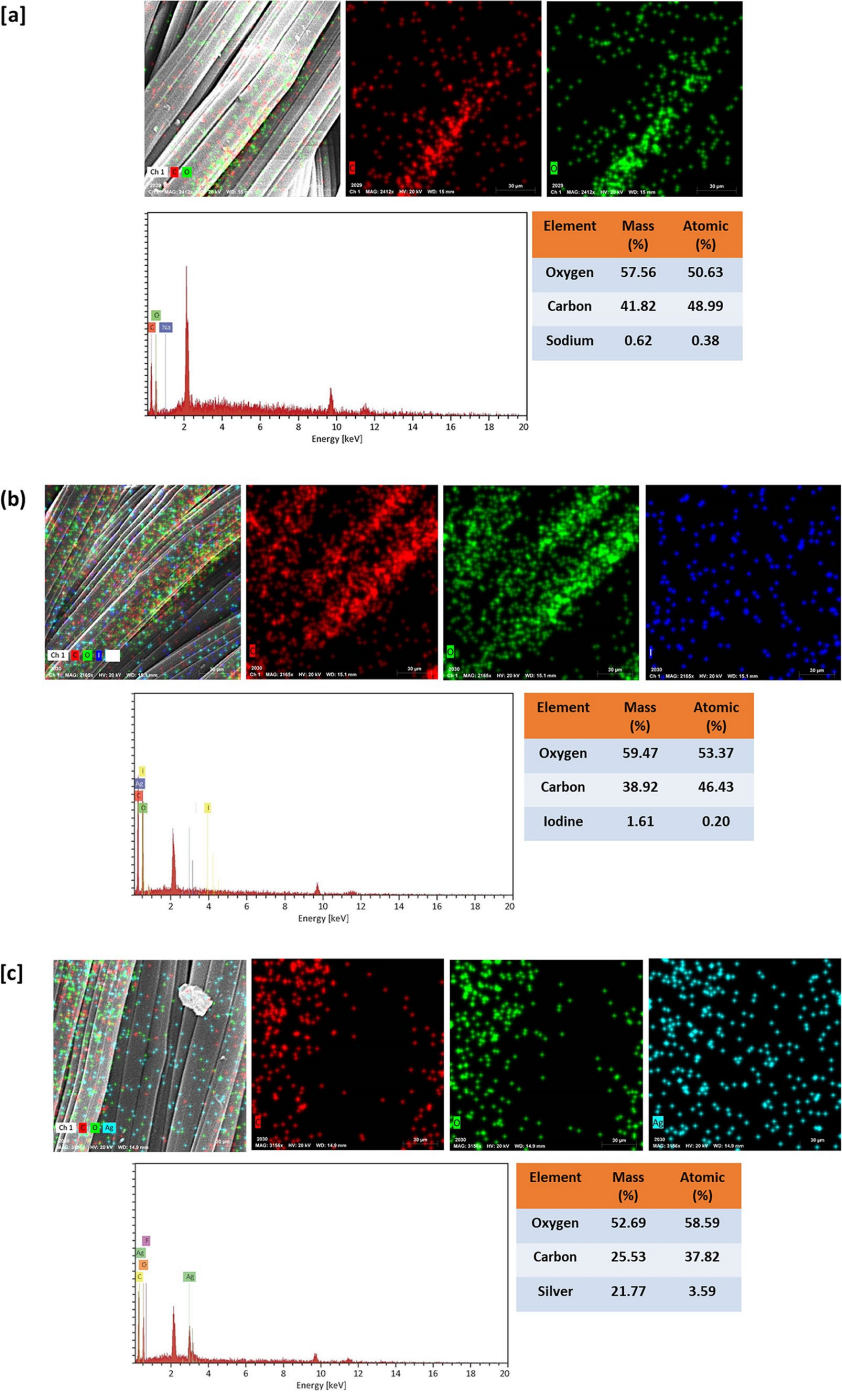

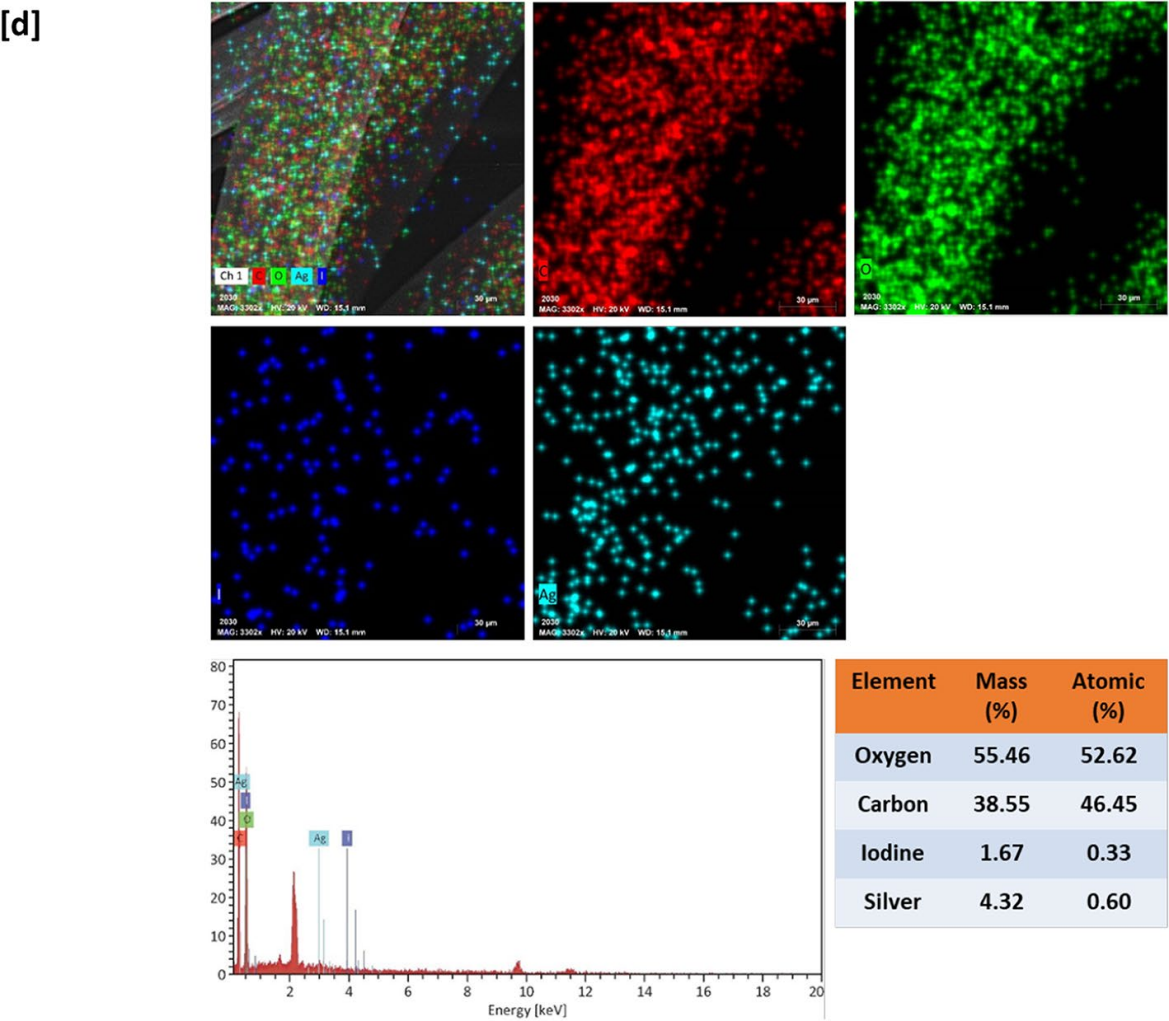
Mapping and energy dispersive X-ray analysis for the bamboo fabric samples; (**a**) bamboo blank, (**b**) AgI@bamboo, (**c**) Ag NPs@bamboo, and (**d**) Ag NPs@AgI@bamboo

The elemental distributions of the bamboo fabrics, AgI@bamboo, Ag NPs@bamboo, and Ag NPs@AgI@bamboo were further examined through energy dispersive X-ray (EDX) analysis. EDX of bamboo fabrics (Fig. 2a) demonstrates the existence of two peaks corresponding to carbon (C) and oxygen (O) related to the elemental components of the bamboo fabrics. Furthermore, elemental mapping was investigated for further confirmation that the surface of bamboo fabrics consists of the C and O. The EDX spectrum of AgI@bamboo (Fig. 2b) and Ag NPs@bamboo (Fig. 2c) reveals a peak at 2 eV caused by I (Fig. 2b) and a peak at 3 eV caused by AgI, (Fig. 2c) in addition to the two bamboo fabric peaks, indicating the existence of iodine and silver nanoparticles on the surface of bamboo fabrics. Furthermore, elemental mapping was investigated for further confirmation of the AgI and Ag presence on the surface of bamboo fabrics. The presence of a peak associated with AgI, Ag, along with the main peaks of bamboo fabrics (C and O elemental), was shown by the EDX of Ag NPs@AgI@bamboo (Fig. 2d), indicating the efficient coating of Ag NPs@AgI on the surface of bamboo fabrics. Meanwhile, the elemental mapping image (Fig. 2d) of Ag NPs@AgI@bamboo shows the differences in color representing different element components (C, O, I, and Ag), inter-composed with Ag NPs@AgI@bamboo with uniform dispersion on the surface, indicating that the Ag NPs@AgI was evenly formed on the bamboo fabric.

**Fig. 3:**
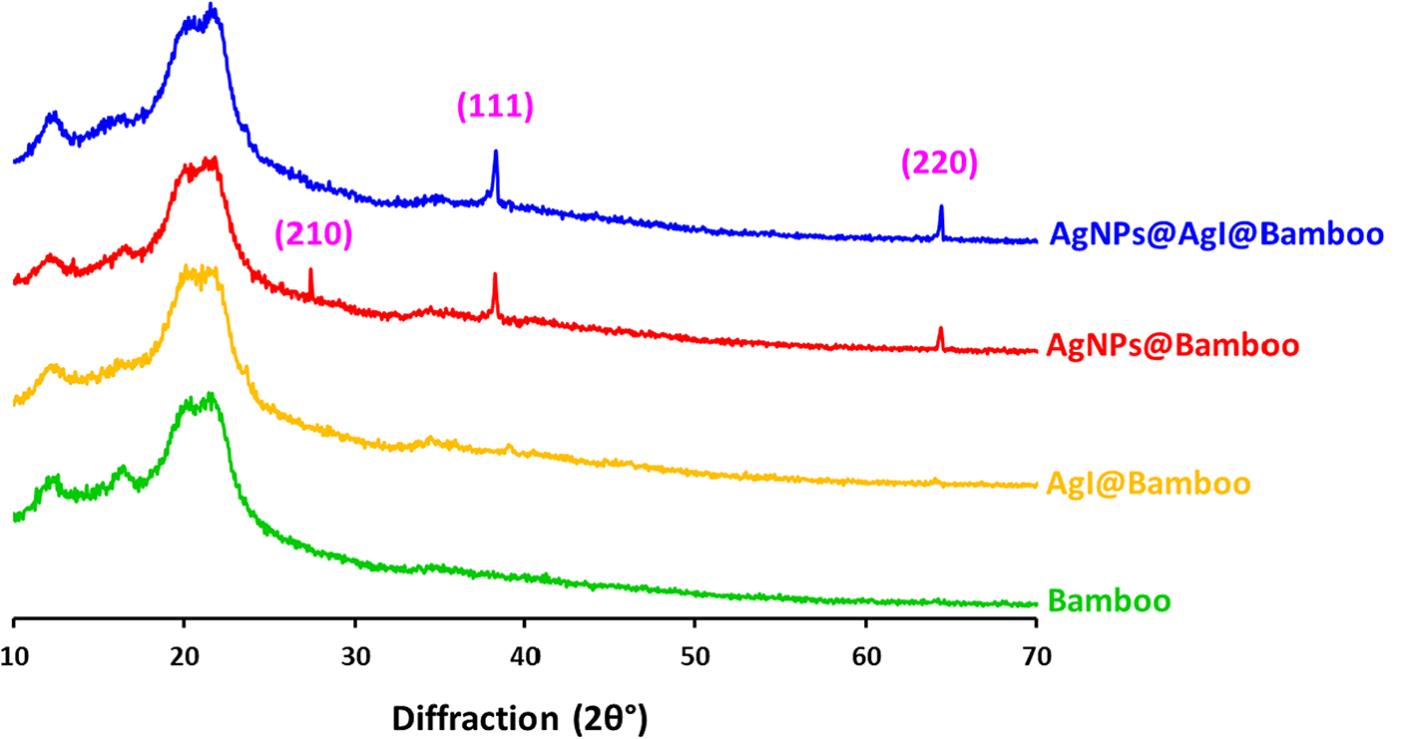
X-ray diffraction patterns for the bamboo fabric samples.

X-ray diffraction (XRD) analysis was performed to display the crystal structure as well as the presence of iodine and silver nanoparticles that were deposited in situ into the fabric surface (Fig. 3). The bamboo fabrics displayed the usual cellulose-II crystalline structure diffraction peaks at 2θ = 12.1°, 16.40°, 21.5°, and 23.3°^76^. XRD results of the AgI@bamboo displayed the same distinctive peaks linked to the crystalline structure of cellulose but with high intensity in the absence of peaks characteristic of β-AgI. The low-intensity peaks of γ-AgI are observed at 2θ = 39.3° and 46.3° being assigned to (110) and (112) γ-AgI, respectively. This means that Ag rich phase of AgI could tend to crystallization in γ-AgI in AgI@bamboo^77^. The XRD pattern of Ag NPs@bamboo shows the cellulose-II crystalline structure. Furthermore, at 2θ angles of 27.6°, 38.3°, and 64.5°, three further diffraction peaks appeared. These were attributed to the (210), (111), and (220) planes of face-centered-cubic Ag^18,78–81^. The XRD pattern of Ag NPs@AgI@bamboo shows the same pattern as Ag NPs@bamboo but is difficult to assign the diffraction peaks of γ- AgI, as their reflections are very close.

**Fig. 4:**
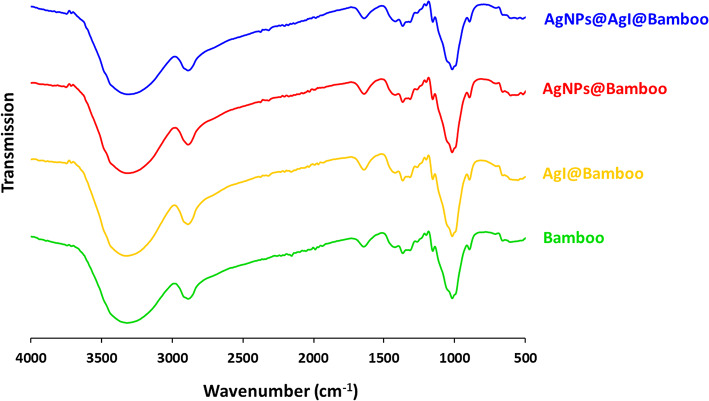
FTIR for the bamboo fabric samples.

The chemical interaction of the AgI, Ag NPs, and Ag NPs@AgI with the surface of the bamboo fabrics was investigated by FTIR. Figure 4 shows the FTIR spectra of the bamboo fabrics, AgI@bamboo, Ag NPs@bamboo, and Ag NPs@AgI@bamboo. FTIR spectrum of the bamboo fabrics shows the characteristic peaks associated with the cellulosic matrix. Bamboo fabrics exhibited distinct peaks that appeared at 3350 cm^− 1^ (O-H stretch), 2900 cm^− 1^ (C-H stretch), 1400 cm cm^− 1^ (C-H wagging), 1440 cm^− 1^ (C-H bending), 1650 cm^− 1^ (C=O stretch), and 1120 cm^− 1^ (C-O stretch)^82^. FTIR spectra of the AgI@bamboo, Ag NPs@bamboo, and Ag NPs@AgI@bamboo showed the same characteristic peaks associated with the bamboo fabrics matrix in the absence of peaks characteristic of AgI, Ag NPs, or Ag NPs@AgI indicating the absence of chemical interactions between these nanoparticles and the functional groups of bamboo fabrics.

**Fig. 5:**
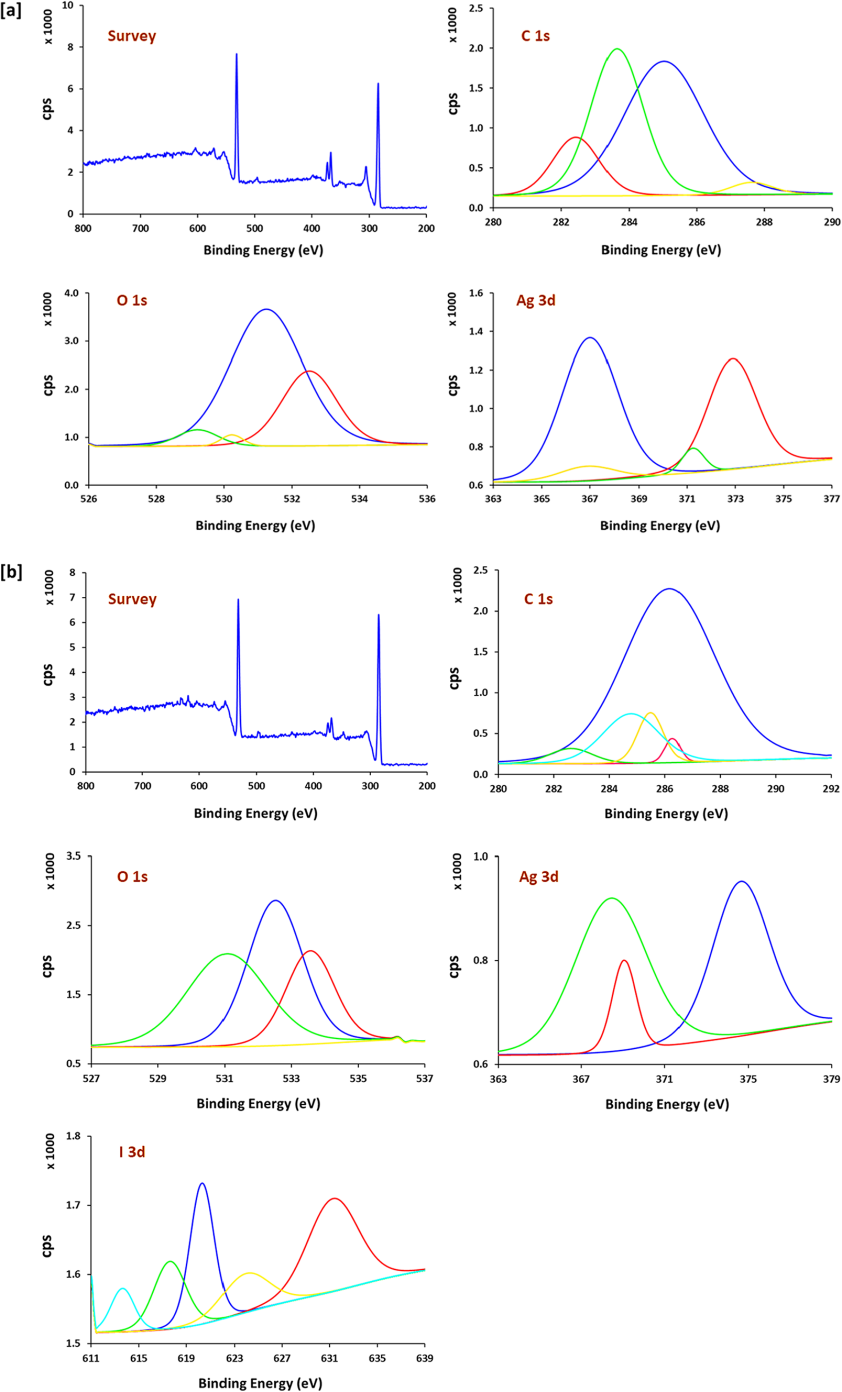
X-ray photoelectron spectra analysis for; (**a**) Ag NPs@bamboo and (**b**) Ag NPs@AgI@bamboo.

To verify the hybridization structure of Ag NPs and Ag NPs@AgI on the surface of the bamboo fabrics, the surface elemental compositions of the Ag NPs@bamboo and Ag NPs@AgI@bamboo were further characterized by XPS. Figure 5a and b show the XPS analysis of the surface element state/chemical composition of the surface of Ag NPs@bamboo and Ag NPs@AgI@bamboo, respectively. The survey spectrum of Ag NPs@bamboo (Fig. 5a) shows the presence of peaks at a binding energy of 532 eV (O 1s) and 284 eV (C 1s) as expected in a spectrum of bamboo fabrics, together with a peak at a binding energy of 368 eV (Ag 3d). The presence of an Ag 3d peak in the XPS spectrum revealed that Ag NPs were successfully deposited in situ on the surface of the bamboo fabrics. The survey spectrum of Ag NPs@AgI@bamboo (Fig. 5b) shows a peak at a binding energy of 619 eV (I 3d) together with three peaks at binding energies of 533 eV (O 1s), 285 eV (C 1s), and 368 eV (Ag 3d). The presence of Ag 3d and I 3d peaks indicates that Ag NPs@AgI were successfully deposited in situ on the surface of the bamboo fabrics.

Bamboo fabrics’ C 1s peak’s XPS high-resolution spectrum displays four C 1s components (C1–C4). C1 is ascribed to the C-C/C-H species of aliphatic and aromatic carbon in lignin; C2 is ascribed to the C-O species (primary and secondary alcohol and ether-type (non-carbonyl group) bonding primarily in bamboo fabrics; C3 is associated with the C = O–O/C − O−C species of carbonyl and acetal groups in bamboo fabrics; and C4 is associated with it^76,83^. The binding energies are estimated at 282–285 eV for C1, 283–287 eV for C2, 284–289 eV for C3, and 288–292 eV for the C4 component. XPS high-resolution spectrum of C 1s of Ag NPs@bamboo (Fig. 5a) showed three peaks (C1-C3). On the other hand, the four peaks (C1-C4) were identified in the XPS high-resolution spectrum of C 1s of Ag NPs@AgI@bamboo (Fig. 5b). C–O–C bond (C3) of Ag NPs@AgI@bamboo is observed to shift to a lower binding energy, which can be explained by the fact that Ag NPs@AgI, as the electron donor, affects the chemical bonding status of the surface. of Ag NPs@AgI@bamboo^16^.

Three components of O 1s (O1-O3) are visible in the high-resolution XPS spectrum of the O 1s peak of bamboo fabrics. O1 is associated with (O-C = O, C = O), which is located at approximately 530–533 eV. O2 is associated with C-O/C − O − C, which appeared at 532–534 eV; and O3 is associated with O-C = O, which is located at 534.536 eV^76^. The XPS high-resolution spectrum of O1s of Ag NPs@bamboo (Fig. 5a) showed three peaks (C1-C3). On the other hand, the four peaks (C1-C4) were identified in the XPS high-resolution spectrum of C 1s of Ag NPs@AgI@bamboo (Fig. 5b).

The valence state of Ag on the surface of Ag NPs@bamboo and Ag NPs@AgI@bamboo was determined by XPS as shown in Fig. 5a and b. Two peaks were seen at 367.1 eV (Ag 3d5/2) and 373.8 eV (Ag 3d3/2) in the Ag 3d peak spectrum of Ag NPs@bamboo ' XPS high-resolution spectrum (Fig. 5a). The successful formation of zero-valent metallic silver (Ag^0^) on the surface of bamboo fabrics was demonstrated by the spin energy separation of Ag 3d, which was 6.7 eV^84^. XPS of the Ag 3d peak spectrum of Ag NPs@AgI@bamboo (Fig. 5b) shows two peaks, Ag 3d3/2 and Ag 3d5/2. Ag 3d5/2 peak was disassembled into two distinct peaks. The peaks at 374.8 eV (Ag 3d_3/2_) and 368.3 eV (Ag 3d_5/2_^)^ were attributed to zero-valent metallic silver (Ag^0^). The peak at 368.7 eV (Ag 3d_5/2_) relates to Ag^+^ assigned to AgI. The successful formation of Ag NPs@AgI on the surface of bamboo fabrics is demonstrated by this result, which demonstrates that the Ag element exists in two forms: Ag + and Ag°^77^.

XPS was used to determine the valence state of I on the surface of Ag NPs@AgI@bamboo. I 3d5/2 and I 3d3/2 have two peaks at 620.3 eV and 630.9 eV, respectively, in the XPS high-resolution spectrum of the I 3d (Fig. 5b). The presence of I in the form of I − assigned to Ag + is indicated by the binding energy of I 3d5/2, which is 620.3 eV. The interstitial iodine and the iodine found in KI residues are responsible for the three peaks that emerged at 613.9, 617.7, and 624.3 eV (3d 5/2)^77^.

**Fig. 6:**
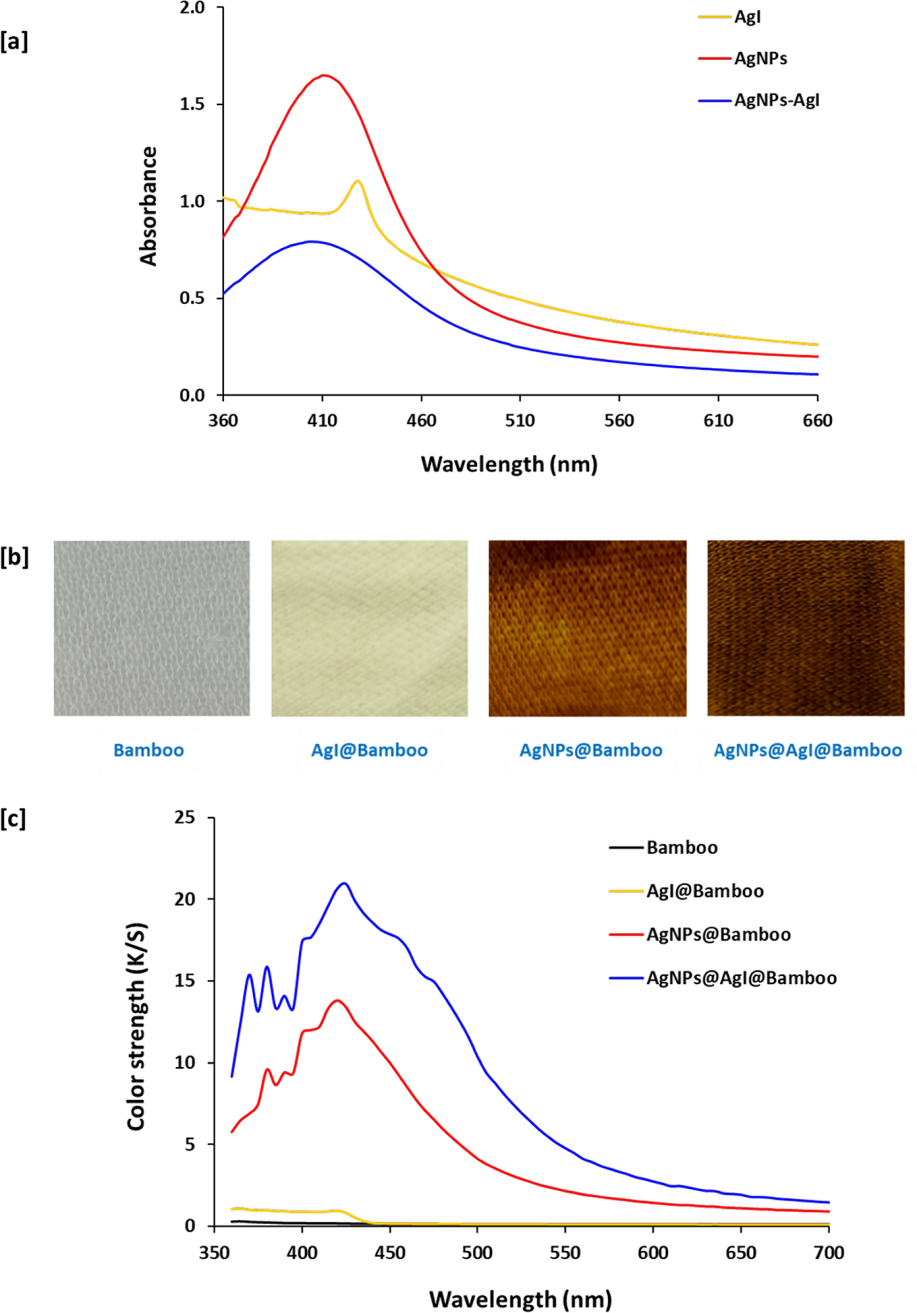
The Optical properties of the bamboo fabric samples; (**a**) the UVUV–visible spectral of the residual solutions; (**b**) the pictures of bamboo fabrics; and (**c**) color strength (K/S).

The UV–-visible spectrum of the residual solutions was studied to confirm the formation of AgI, Ag, and Ag NPs@AgI on the surface of bamboo fabrics. Figure 6a shows the UV–visible spectrum of the residual solutions. This figure reveals that the appearance of a surface Plasmon resonance (SPR) above 400 nm confirms the formation of nanoparticles on the surface of bamboo fabrics^85^. The residual solution of AgI@bamboo exhibited an exciting peak that appears at 425 nm, with a knee which is attributed to γ-AgI, indicating the formation of AgI on the surface of bamboo fabrics^86^. This result is in harmony with that obtained by the XRD of AgI@bamboo. The residual solution of Ag NPs@bamboo exhibited a strong absorption peak with high intensity at 410 nm, attributed to the LSPR of Ag^0^, which is a typical absorption peak of spherical Ag NPs due to their surface Plasmon, indicating the formation of Ag NPs on the surface of bamboo fabrics^87^. On the other hand, the residual solution of Ag NPs@AgI@bamboo shows an abroad, strong absorption peak with red shifting of the LSPR, attributing to the dielectric constant of the AgI surface.

Figure 6b shows the pictures of the bamboo fabrics AgI@bamboo, Ag NPs@bamboo, and Ag NPs@AgI@bamboo. This figure shows that the color of the bamboo fabric is white. The color of the AgI@bamboo is pale yellow due to the deposition of AgI^57^. The color of the Ag NPs@bamboo is yellow/brownish due to the deposition of silver nanoparticles, and the color of Ag NPs@AgI@bamboo is dark brown due to the deposition of silver nanoparticles as well as AgI.

Figure 6c shows the K/S values of bamboo fabrics AgI@bamboo, Ag NPs@bamboo, and Ag NPs@AgI@bamboo. This figure shows that the K/S values of the bamboo fabrics are close to 0 when the wavelength changes from 350 to 700 nm. K/S values of the AgI@bamboo are slightly higher than those of the bamboo fabrics. Unlike both samples, the K/S values of Ag NPs@bamboo and Ag NPs@AgI@bamboo are much higher than those of the bamboo fabrics and AgI@bamboo. The significant increment of K/S values for Ag NPs@AgI@bamboo compared to Ag NPs@bamboo indicates better color strength of Ag NPs@AgI@bamboo.

Table 1 shows the colorimetric measurements, including CIE (L^*^, a^*^, b^*^), and K/S of bamboo fabrics AgI@bamboo, Ag NPs@bamboo, and Ag NPs@AgI@bamboo. The original color of bamboo fabrics is white; with a high L^*^ value (83.09 ± 1.00), a negative a^*^ value (-0.29 ± 0.00), and a low b^*^ value (2.66 ± 0.17). Depending on the exploited nanoparticles, AgI@bamboo, Ag NPs@bamboo, and Ag NPs@AgI@bamboo were exhibited with significant differences in the estimated L^*^, a^*^, and b^*^ values compared to the blank one. However, an insignificant change occurred in the L^*^ value between bamboo fabrics and AgI@bamboo. AgI@bamboo showed an increment in negative a^*^ value, which indicates a greener color, and an increase in positive b^*^ value, which indicates a more yellow color.

On the contrary, the lightness (L^*^) of Ag NPs@bamboo and Ag NPs@AgI@bamboo is significantly lower than that of bamboo fabrics due to the deposition of Ag NPs and AgI on the surface of bamboo fabrics. However, an insignificant change occurred in L^*^ value between Ag NPs@bamboo and Ag NPs@AgI@bamboo. In the case of Ag NPs@AgI@bamboo, a considerable decrease in the L^*^ value indicates a darker hue upon the formation of Ag/AgI on the fabric surface. The increased positive a^*^ values indicate a redder color, and the increased positive b^*^ values indicate a more yellow color. Thus, the color of the bamboo fabric changes from white to yellow/brownish and dark brown after the deposition of Ag NPs and Ag NPs@AgI, respectively.

**Table 1:** Color coordinates data for the bamboo fabric samples.

Sample	L^*^	a^*^	b^*^	C^*^	h^o^	K/S
Bamboo blank	83.09 ± 1.00	-0.29 ± 0.00	2.66 ± 0.17	2.68 ± 0.18	96.24 ± 0.54	0.17 ± 0.02
AgI@bamboo	83.16 ± 0.36	-3.0 ± 0.01	9.40 0.53	9.87 ± 0.50	107.74 ± 0.94	0.94 ± 0.06
Ag NPs@bamboo	47.32 ± 3.83	7.70 ± 1.00	34.25 ± 2.67	35.11 ± 2.80	77.36 ± 0.86	13.81 ± 2.95
Ag NPs@AgI@bamboo	36.26 ± 1.44	12.70 ± 0.84	29.09 ± 1.67	31.74 ± 1.74	66.40 ± 1.25	20.65 ± 1.25

### Antimicrobial activities of coated cotton fabrics

**Fig. 7:**
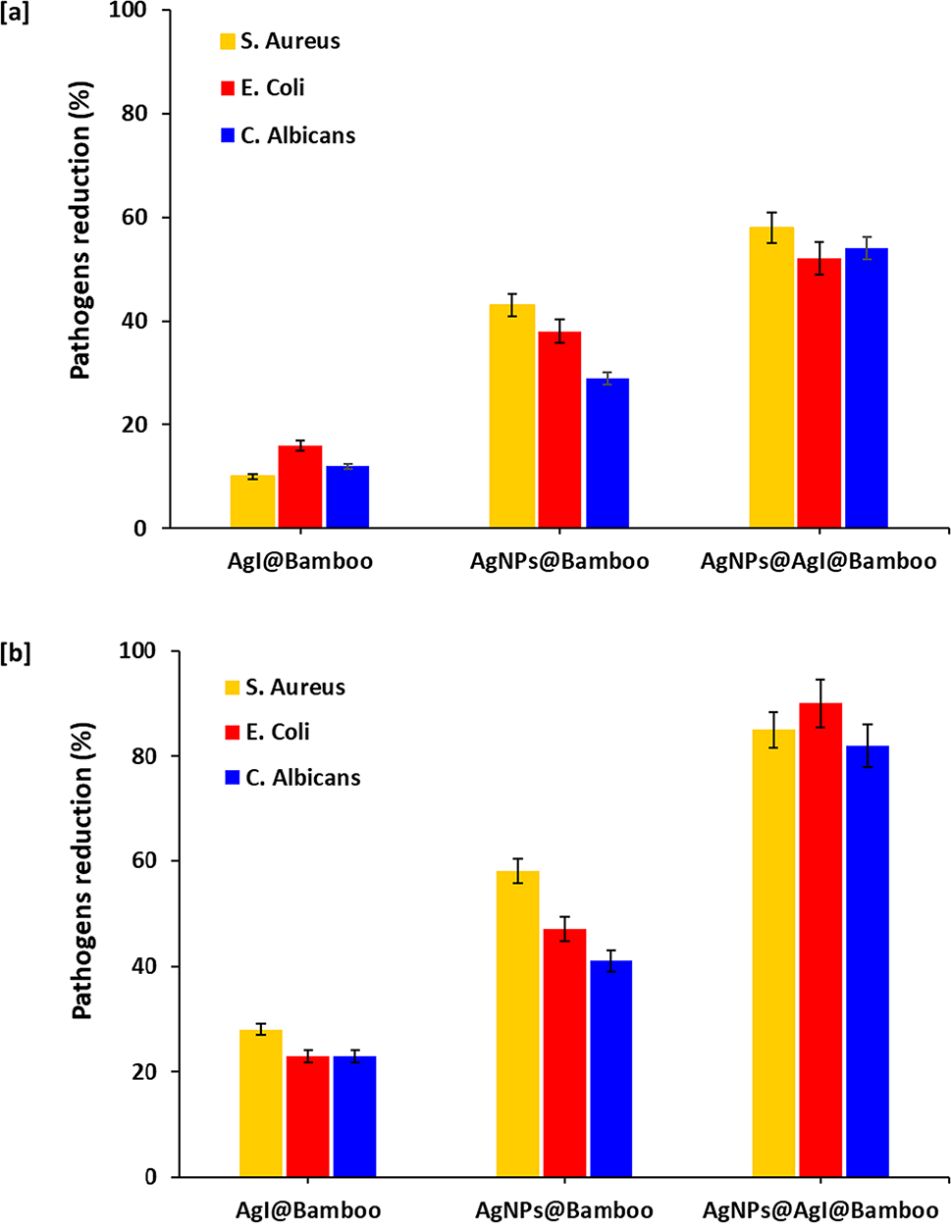
Antimicrobial results for the bamboo fabric samples; (**a**) dark light and (**b**) UV light irradiation.

The antimicrobial photocatalytic inactivation performances were evaluated against three microorganisms, including *S. aureus* (a Gram-positive bacterium), *E. coli* (a Gram-negative bacterium), and *C. albicans* (a fungus). Figure 7 shows the pathogen’s reduction of microorganisms, and suspension after 1-hour contact with AgI@bamboo, Ag NPs@bamboo, and Ag NPs@AgI@bamboo under dark light (Fig. 7a) and UV light irradiation (Fig. 7b). The non-microbicide effect of bamboo fabrics and the lack of any effect from UV light irradiation were demonstrated by the lack of a discernible drop in the population of microbes when the fabrics were exposed to either dark light or UV light. Unlike the bamboo fabrics, the AgI@bamboo, Ag NPs@bamboo, and Ag NPs@AgI@bamboo samples exhibited antimicrobial activities, with different pathogen reduction percentages.

Under dark light, the antimicrobial activities of the AgI@bamboo sample were relatively low, not exceeding 10%, 18%, and 15% for *S. aureus*, *E. coli*, and *C. albicans*, respectively, indicating a fairly low microbial inactivation effect. The antimicrobial activities of the AgI@bamboo sample were enhanced under UV light irradiation against tested microorganisms, with pathogen reduction percentages of 30, 25, and 26 for *S. aureus*, *E. coli*, and *C. albicans*, respectively. The AgI@bamboo sample showed good antimicrobial activities under dark and UV light irradiation. Under dark light, the pathogen reduction was 43%, 39%, and 30% for ***S. aureus***, *E. coli*, and *C. albicans*, respectively. However, under UV light irradiation, the pathogen reduction increased to 59%, 55%, and 43% for *S. aureus*,* E. coli*, and *C. albicans*, respectively. Ultimately, the Ag NPs@AgI@bamboo sample showed the highest antimicrobial activities under dark and UV light irradiation. Under dark light, Ag NPs@AgI@bamboo samples showed very good antimicrobial activities with pathogen reductions of 60%, 58%, and 59% for *S. aureus*,* E. coli*, and *C. albicans*, respectively. Under UV light irradiation, the Ag NPs@AgI@bamboo sample showed excellent antimicrobial activities with pathogen reductions of 88%, 90%, and 82% for *S. aureus*, *E. coli*, and *C. albicans*, respectively. This high enhancement of the microbial inactivation suggests that the photocatalytic process is primarily responsible for the microbial activity of the microbial activity of Ag NPs@AgI@bamboo. The intrinsic disinfection, a characteristic of the deposited Ag NPs, is the second factor contributing to this enhancement.

In summarization, it could be pointed out that (1) the microbial effect of the modified bamboo fabrics is strongly activated by light, meaning that the microbial inactivation is driven by a photochemical mechanism; (2) the presence of silver nanoparticles (Ag NPs) strongly boosts the microbial inactivation, even when present in a low amount. (3) The inclusion of AgI seemingly did not contribute to further enhancing the bactericidal effect of modified cotton.

Several studies were reported to investigate the antimicrobial activity of Ag NPs and Ag/AgX nanostructures. The release of Ag^+^, which reacts with the -SH groups of proteins to render them inactive, is typically assumed to be the antimicrobial mechanism of Ag NPs. Moreover, the bacterial cell wall and membrane may be penetrated by the released Ag+, leading to cell death. Typically, Ag/AgX nanostructures demonstrate three different antimicrobial mechanisms: (i) the release of Ag+, which denatures proteins and decreases the permeability of bacterial cell membranes. (ii) By exposure to light, Ag/AgX nanostructures produce active species like reactive oxygen species (ROS) and reactive halogen species (RHS), which harm the cell walls and membranes and undermine bacteria’s defenses. (iii) Ag/AgX nanostructures can enhance light absorption and create new opportunities for the development of photo-catalysts that are exposed to light.

Ag NPs@AgI@bamboo exhibit higher photocatalytic activity than AgI NPs@bamboo and Ag NPs@bamboo. The release of Ag^+^, which is known to cause protein denaturation and cell death, could account for the microbial effects seen in the dark. There are two categories for the Ag NPs@AgI@bamboo sample’s synergistic antimicrobial mechanism under light. First, in the presence of light, the liberated Ag^+^ reacts with the generation of ROS. Ag^+^ can change the permeability of cell membranes and denaturant bacterial proteins, while ROS produced by photocatalysts can effectively eradicate bacteria when exposed to light^88^.

### Removal of methylene blue dye

#### Catalytic activity (decolorization of MB)

**Fig. 8:**
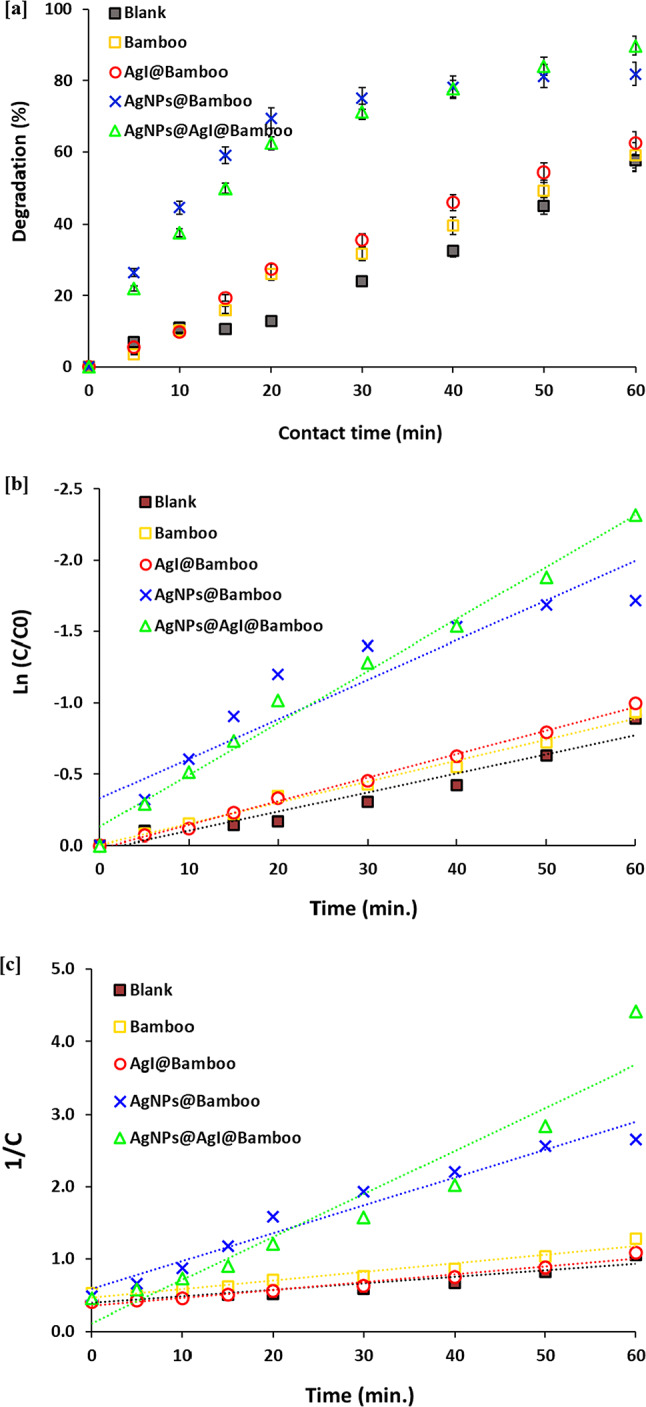
Catalytic activity results for the bamboo fabric samples; (**a**) catalytic percentage of MB, (**b**) Comparison between adsorption (without NaBH4) and catalytic degradation (with NaBH4) for Ag NPs@AgI@Bamboo and (**c**) First-order kinetic and (**e**) Second-order kinetic.

Methylene blue (MB) is selected as the model dye to investigate the catalytic and photocatalytic activity of AgI@bamboo, Ag NPs@bamboo, and Ag NPs@AgI@bamboo samples. Bamboo fabrics, AgI@bamboo, Ag NPs@bamboo, and Ag NPs@AgI@bamboo samples were employed for sodium borohydride (NaBH_4_) based catalytic degradation of MB dye. The catalytic reduction of methylene blue (MB) to leucomethylene blue (LMB) can be monitored with UV-vis absorption through its color change from blue to colorless, The MB molecule exhibits two distinct absorption peaks at 665 and 608 nm, which are caused by the п → п^*^ and n → п^*^ transitions. The catalytic reduction can be observed through the gradual decrease in the absorbance peak at 665 nm with the progress of time, as well as by the naked eye through a gradual change in the blue color of MB into a colorless LMB. The catalytic reduction of methylene MB by NaBH_4_ was investigated in the absence of fabric samples. Figure 8a shows the catalytic % of MB dye with time in the presence of different fabric samples.

These figures reveal that the absorption peak of MB gradually decreases as the reaction time increases. In the absence of fabric samples (blank sample), a very small decrease in the absorption peak was observed, and this characteristic peak reduced slowly with time. The catalytic % of MB dye by NaBH_4_ was 58 after 60 min. This result shows that the reduction of MB with only NaBH4 is extremely slow. The redox potential of methylene blue is 0.5 V, and that of NaBH_₄_ is − 1.24 V. Therefore, the reduction of methylene blue with NaBH_₄_ is thermodynamically favorable. However, there is a kinetic barrier that prevents this reaction. This may be caused by the large potential difference between donor and acceptor molecules. Bamboo fabrics and AgI@bamboo showed the same trend as the blank but with a little higher catalytic % of MB. The catalytic % of MB dye by bamboo fabrics and AgI@bamboo was 61% and 62%, respectively, after 60 min. This result may be attributed to the physical absorbing capacity of the bamboo fabrics. Bamboo fabrics contain many hydroxyl groups, facilitating the physical adsorption of MB via van der Waals forces, enabling the removal of dye. Compared with the bamboo fabrics and AgI@bamboo, Ag NPs@bamboo, and Ag NPs@AgI@bamboo samples showed better catalytic activity. A very sharp decrease in the absorption peak was observed, and this characteristic peak reduces highly with time. Ag NPs@bamboo and Ag NPs@AgI@bamboo samples exhibited better catalytic activity with a catalytic removal efficiency of 85% and 90%, respectively, after 60 min. The enhancement of the catalytic activity of Ag NPs@bamboo and Ag NPs@AgI@bamboo may be attributed to the positive effect of Ag NPs on the acceleration of the reduction reaction. This positive effect can be attributed to two reasons: (i) the larger specific area of fabric catalyst increases the collision chance between molecules once the BH_4_– is absorbed on the surface of fabrics; (ii) Ag NPs function as an electron relay, triggering the electron transformation from BH_4_– ions (donor B_2_H_4_/BH_4_^–^) into the acceptor (leuco-MB, referring to the reduced MB/MB), leading to more potent dye reduction. The increase in the catalytic activity of the Ag NPs@AgI@bamboo sample compared to the Ag NPs@bamboo sample could be attributed to the availability of catalytic active sites such as Ag NPs and AgI on its surface.

For further confirmation of the efficiency of the catalytic degradation of MB dye, adsorption experiments without NaBH_4_ (as adsorption) were carried out, while the results were presented in the supplementary data (Figure S1). The data showed that the removal of MB dye by adsorption was quite low and insignificant compared with catalytic degradation. For Ag NPs@AgI@bamboo, the removal percentage by adsorption was 8.7% within 60 min; however, the catalytic degradation reached 89.8% in the case of catalytic degradation. These data reflected that the adsorption is not significantly effective in the removal of MB at the applied condition, while the catalytic degradation of MB dye was quite efficient by using Ag NPs@AgI@bamboo.

Figure 8b shows the pseudo-first-order fitting data for the MB catalytic reduction in the absence and presence of bamboo fabric samples. Figure 8c shows the pseudo-second-order fitting data of the MB catalytic reduction in the absence and existence of bamboo fabric samples. The pseudo-first-order parameters were calculated using the linear plot of ln C/C^o^ over time. The parameters of the pseudo-second-order model were determined from the linear plot of 1/C vs. time. Following their evaluation of the two models, the kinetic parameters of the two models are presented in Table 2. Table 2 shows the coefficient of determination (R²), reaction rate constant (k), and the catalytic half-time (t_1/2_) of the two models. This table reveals that the R^2^ values of pseudo-first-order are higher than those of pseudo-second-order, and these values are within the range of 0.993–0.998 for the fitted lines, indicating a good linear correlation and demonstrating that MB reduction has a pseudo-first-order reaction rate. The pseudo-first-order model shows that the rate of the catalytic reduction reaction is dependent on the MB concentration and independent of the sodium borohydride.

The reaction rate constant was 0.0643, 0.0717, 1.0207, and 1.0581 min^− 1^ for bamboo fabrics, AgI@bamboo, Ag NPs@bamboo and Ag NPs@AgI@bamboo, respectively. The results show that bamboo fabrics, and AgI@bamboo, could catalyze the reduction of MB, but with slower reaction rates than Ag NPs@bamboo and Ag NPs@AgI@bamboo. The reaction rate constant value in the case of Ag NPs@AgI@bamboo catalyst is higher by 1.5 and 2.5 times than Ag NPs@bamboo and bamboo fabrics, respectively. This result illustrates the impact of the AgI nanoparticles in speeding up the catalytic reduction rate of the MB dye.

The half-life (t_1/2_) of catalytic reduction was 107.84, 96.73, 39.77, and 33.04 min for bamboo fabrics, AgI@bamboo, Ag NPs@bamboo, and Ag NPs@AgI@bamboo, respectively. The results indicate that Ag NPs@AgI@bamboo showed the shortest half-reduction time compared to other bamboo fabric samples. This result indicates that the catalytic reduction of MB in the existing Ag NPs@AgI@bamboo catalyst is faster by 3.1 times than that in the presence of bamboo fabrics. The multi-step process of reducing MB with NaBH4 in the presence of catalysis involves both physical and chemical mechanisms. The MB dye molecules adsorb on the catalytic surface due to van der Waals forces between the two molecules. The MB dye molecules then undergo the transfer of electrons from NaBH4 to them, transforming them into their reduced state. The Ag NPs catalyze this process by providing a suitable surface for the adsorption of MB dye molecules and promoting the electron transfer reaction. The MB decrease in the LMB process can be explained by the following reactions (Eqs. 5, 6).

5 $${\text{BH}}_{4}^{ - } + {\text{2H}}_{{\text{2}}} {\text{O}} \to {\text{BO}}_{2}^{ - } + {\text{ 4H}}_{{\text{2}}}$$

6 $${\text{BH}}_{4}^{ - } + {\text{4MB}}^{ + } + {\text{2H}}_{{\text{2}}} {\text{O}} \to {\text{BO}}_{2}^{ - } + {\text{4LMB}} + {\text{4H}}^{ + }$$

Reaction (1) (Eq. 5) might occur in bulk solution as a homogeneous reaction, while a heterogeneous reaction could perform on the catalytic surfaces. The primary reaction that only takes place on catalytic surfaces is Reaction 2 (Eq. 6)^89,90^. MB undergoes a two-electron reduction reaction to LMB. The reduction of the double bond in the heterocyclic ring causes p-conjugation to break and the electron delocalization length of MB to decrease. NaBH_4_ then transfers two electrons to MB^+^. The BH_4_^−^ions from NaBH_4_ function as a nucleophile and MB as an electrophile in the heterogeneous catalyst reactions of MB dye decolorization with silver nanoparticles in the presence of NaBH_4_. First, a straightforward electrostatic interaction causes BH_4_^−^ions and MB dye to be adsorbed on the Ag NPs@AgI@bamboo catalyst surface. Because of the increased electron density brought on by the excess of BH_4_^−^ adsorbed on the catalytic surface, the metal’s Fermi level rises, transferring electrons to the dye molecules and ultimately reducing them to lecuomethylene blue (LMB). Because of their high negative potential (-1.8 V vs. NHE), Ag NPs act as electron relays, facilitating the transfer of electrons from the BH4-ion toward the MB dye. Protons are produced and electrons are released onto the Ag surface by the BH4-ions. The Ag nanoparticles absorb the electrons, which are subsequently transferred to the adsorbed MB. The colorless Leuco-methylene blue (LMB) is produced when the adsorbed MB dye molecules are reduced after accepting electrons from the Ag NPs@AgI@bamboo catalyst’s surface. The MB molecule’s C = N bonds are changed into -NH- bonds to create the LMB, a less hazardous byproduct. The LMB, as a less toxic product, is formed by the transformation of the C = N bonds of the MB molecule into –NH– bonds. Following that, the reaction products are desorbed into the aqueous solution from the catalyst surface^91^.

### Photocatalytic activity

**Fig. 9:**
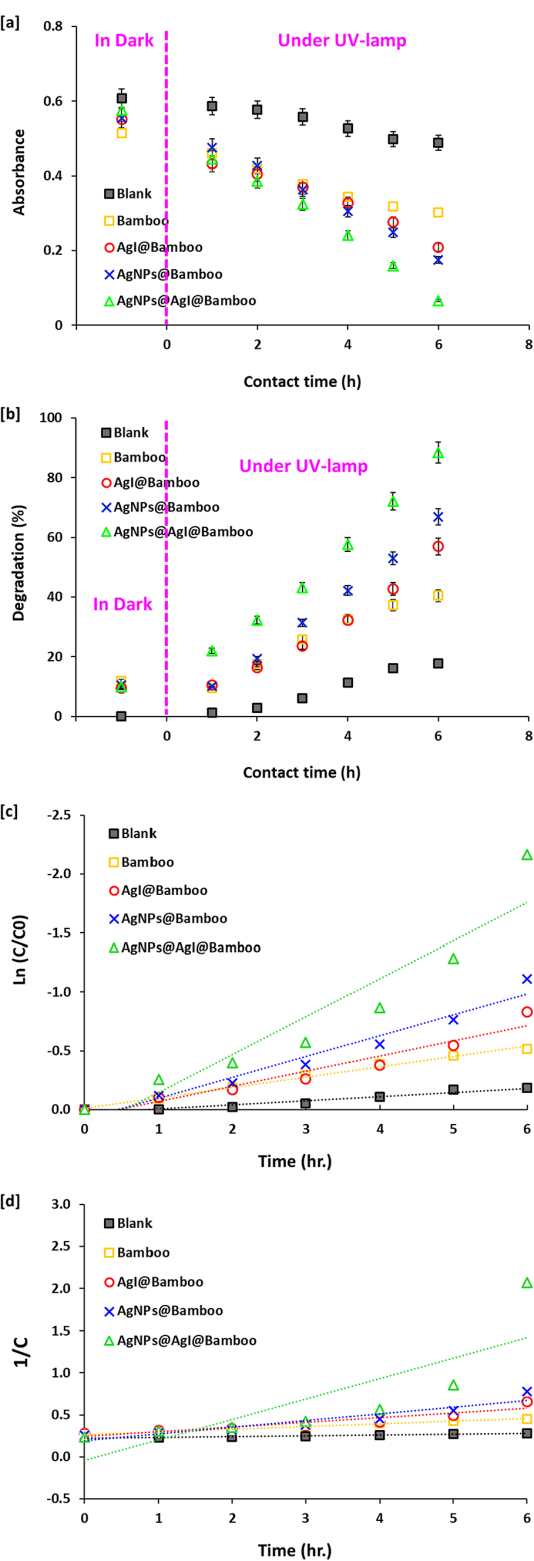
Photocatalytic activity results in the bamboo fabric samples; (**a**) absorbance of residual MB, (**b**) degradation percentage of MB, (**c**) First-order kinetic and (**d**) Second-order kinetic.

The photocatalytic degradation performances of the bamboo fabrics, AgI@bamboo, Ag NPs@bamboo, and Ag NPs@AgI@bamboo samples were investigated in an aqueous solution of MB dye under the irradiation of UV light. The efficiency of MB degradation was evaluated by monitoring the changes in the UV-visible absorbance at 665 nm throughout time. Figure 9a shows the absorption spectra of MB dye at different degradation times, in the presence of different fabric samples. Figure 9b shows the photocatalytic degradation % of MB dye with time in the presence of different fabric samples. These figures demonstrate that the intensity of the absorption peak at 665 nm decreased with an increase in the UV irradiation time (Fig. 9a).

Additionally, for further confirmation of the effective removal of MB dye by photocatalytic degradation, experiments in the dark (as adsorption) were performed, while the data were shown in the supplementary data (Figure S2). The results showed that the removal of MB dye by the adsorption process was insignificant compared to the photocatalytic degradation. Within 1 h, the adsorption percentage of MB onto all applied fabrics ranged from 9.5 to 11.9% only. At using Ag NPs@AgI@bamboo, the removal of MB in 1 h by adsorption was 9.8%, while the photocatalytic degradation was 21.9% and reached 88.4% after 6 h. The data declared that the photocatalytic degradation of dye at the applied condition was highly efficient compared with the adsorption.

The photocatalytic degradation was increased with prolonged UV irradiation time (Fig. 9b). For Ag NPs@AgI@bamboo, the characteristic absorption peak at 665 nm was decreased rapidly with the increase of irradiation time. This decrease in absorbance reflects the photocatalytic degradation of MB on the surface of Ag NPs@AgI@bamboo. Neither the blank photolysis (without a fabric photocatalyst) nor the photocatalytic experiment (with bamboo fabrics) showed any noticeable improvement in the decrease in the MB concentration with the interval time. The MB concentration without photocatalytic remained (direct photolysis of MB) almost at the same level under the effect of UV irradiation, with an insignificant decrement in MB degradation (12%). This is likely due to the interaction of MB with light, which means that MB is stable under UV light; so the photolysis of the MB dye is negligible. The photocatalytic degradation rate of MB in contact with bamboo fabrics was very low (35%), indicating the bamboo fabric itself does not possess any photocatalytic activity. The insignificant decrement in MB concentration for the bamboo fabric is most likely due to the adsorption of MB on the bamboo fabric surface The photocatalytic degradation efficiency of the treated bamboo fabric samples followed the descending order of Ag NPs@AgI@bamboo > AgI@bamboo > Ag NPs@bamboo. Ag NPs@bamboo demonstrate comparatively lower photocatalytic activity, with a degradation efficiency of 50%, compared to AgI@bamboo, which demonstrates a degradation efficiency of 65% in 6 h under UV light irradiation. After absorbing a photon, the AgI produces an electron to reduce the Ag^+^ ion to give the Ag^0^ atom. However, AgI is not suitable as a photocatalytic material because of the light instability.

In comparison with AgI@bamboo and Ag NPs@bamboo, Ag NPs@AgI@bamboo showed the highest photocatalytic activity. This result, therefore, confirmed that combining silver nanoparticles (Ag NPs) with silver iodide (AgI) acted in the enhancement of the photocatalytic activity, leading to 93% degradation of MB within 6 h under UV light irradiation. The formation of Ag^0^ on the AgI surface causes a dual interaction with light; Ag_0_ NPs display SPR, enhancing the light absorption of the Ag@AgI. Second, Ag^0^ NPs allow for charge separation between electrons (e^−^) and holes (h^+^). The excellent photocatalytic activity of Ag NPs@AgI@bamboo may be referred to the advantageous effects of the Ag-NPs generated on the AgI@bamboo:

To further investigate the reaction mechanism, pseudo-first- and pseudo-second-order kinetics models were tested. Figure 9c depicts the pseudo-first-order fitting results for MB photocatalytic degradation in the presence and absence of bamboo fabric samples. Figure 9d depicts the pseudo-second-order fitting results for MB photocatalytic degradation in the absence and presence of bamboo fabric samples. The parameters of the pseudo-first-order parameters were evaluated from a linear plot of ln C/C_o_ over time and the parameters of the pseudo-second-order model were calculated using a linear plot of 1/C vs. time, Table 2 shows the kinetic characteristics of the two models, Table 2 displays the coefficients of determination (R^2^), reaction rate constant (k), and photocatalytic half-life (t_1/2_) for both models.

This table shows that the pseudo-first-order R^2^ values in this table are higher than the pseudo-second-order R^2^ values, and they fall between 0.996 and 0.999 for the fitted lines, indicating a good linear correlation and confirming that MB photocatalytic degradation has a pseudo-first-order rate. The pseudo-first-order model indicates that the rate of the photocatalytic degradation reaction is dependent on the MB concentration. For bamboo fabrics, AgI@bamboo, Ag NPs@bamboo, and Ag NPs@AgI@bamboo, the corresponding reaction rate constants were 0.3843, 0.5588, 0.7712, and 1.3977 min^− 1^. The reaction rate constant value in the case of Ag NPs@AgI@bamboo is higher by 3.6 times higher than that in bamboo fabrics. The photocatalytic degradation half-lives (t_1/2_) were 1082.02, 744.05, 539.18, and 297.48 min for bamboo fabrics, AgI@bamboo, Ag NPs@bamboo, and Ag NPs@AgI@bamboo, respectively. The results demonstrate that Ag NPs@AgI@bamboo have the quickest half photocatalytic degradation time when compared to other bamboo fabric samples. These findings reveal that the photocatalytic degradation of MB by the Ag NPs@AgI@bamboo catalyst is 3.5 times faster than that in the presence of bamboo fabrics.

The proposed photocatalytic mechanism may be a type-II mechanism with AgI as a mediator for the electron’s transference. AgI has a band gap of 2.8 eV, and its valence band (VB) and conduction band (CB) edge potentials are positioned at 2.38 eV and − 0.42 eV (vs. NHE), respectively. The surface Plasmon resonance (SPR) of Ag-NPs allows the Ag NPs@AgI to be stable and efficient under light. Under UV light irradiation, Ag-NPs can create electrons (e^−^) in the conduction band (CB) and holes (h^+^) in the valence band (VB) via SPR. The AgI was also excited by the UV light to generate **e −** and h^+^. The electrons in Ag NPs could be transported to the CB of the AgI. AgI can operate as electron traps, capturing a certain amount of electrons and increasing the separation between e^−^ and h^+^. The electrons at CB of AgI could be transferred to the present molecular oxygen (O_2_) to form the active species oxidant (O_2_^•−^), which can assist the degradation of MB. Conversely, the VB of AgI and the surface of Ag NPs both have holes (h^+^) left in them. The h^+^ can undergo oxidation from I^−^ to I^0^, which are strong oxidizing agents capable of attacking MB and returning to their ionic I^−^ state. During the process of photocatalytic degradation of MB, I^0^ atoms are reduced to Iˉ ions again, and Iˉ ions and Ag^+^ ions are combined to form AgI and maintain the self-stability of the system. Furthermore, surface-adsorbed H_2_O and OHˉ can be oxidized by h^+^ in the VB of AgI to create ^•^OH radicals. The OH ^•^OH radicals can readily degrade MB into CO_2_ and H_2_O by oxidization^57,92^.

**Table 2:** Kinetic parameters for catalytic and photocatalytic degradation of MB dye in the presence of the bamboo fabric samples.

	Sample	First-order			Second-order		
		K_2_ × 10^− 3^ (1/sec.)	t_1/2_ (min)	R^2^	K_2_ × 10^− 3^ (1/M.sec.)	t_1/2_ (min)	R^2^
Catalytic	Bamboo blank	6.43	107.84	0.99	3.08	169.49	0.95
	AgI@Bamboo	7.17	96.73	0.99	2.21	113.41	0.97
	Ag NPs@Bamboo	12.07	39.77	0.99	9.28	43.59	0.95
	Ag NPs@AgI@Bamboo	15.81	33.04	0.99	13.46	31.82	0.96
Photocatalytic	Bamboo blank	38.43	1082.02	0.99	4.25	3703.70	0.99
	AgI@Bamboo	55.88	744.05	0.99	7.88	3692.31	0.98
	Ag NPs@Bamboo	77.12	539.18	0.99	10.26	2569.59	0.95
	Ag NPs@AgI@Bamboo	139.77	297.48	0.99	29.22	1562.50	0.94

## Conclusion

Herein, the multi-functional bamboo fabrics were designed for the disposal of complex wastewater, including pathogenic microorganisms and dyes from wastewater. Here, the surface of bamboo fabrics was modified by the decoration of its surface with AgI, Ag NPs, and Ag NPs@AgI via the in situ method. The efficiency of bamboo fabrics and decorated bamboo fabrics with AgI, Ag NPs, and Ag NPs@AgI was evaluated in challenging environmental applications by studying the removal of different microorganisms and methylene blue as model dye. Ag NPs@AgI@bamboo showed the highest pathogenic microorganism removal %. The efficiencies of the Ag NPs@AgI@bamboo were 88, 90, and 82% under UV light irradiation compared to 60, 58, and 59% under dark conditions towards *S. aureus*, *E. coli*, and *C. albicans*, respectively. Ag NPs@AgI@bamboo showed the highest reduction of MB (90%) compared to 62% and 85% for AgI@bamboo and Ag NPs@bamboo, respectively, after 60 min. Ag NPs@AgI@bamboo displayed the highest photodegradation % of MB (93%) compared to 65% and 50% for AgI@bamboo and Ag NPs@bamboo, respectively, after 6 h under UV light irradiation. This study showed that combining the plasmonic effect of noble nanoparticles (Ag NPs) with silver iodide (AgI) immobilized on the substrate surface (bamboo fabrics) can result in Ag NPs@AgI@bamboo, which have excellent antimicrobial, catalytic, and photocatalytic properties and are easily recyclable by removing them from the solution with tweezers.

## Electronic supplementary material

Below is the link to the electronic supplementary material.

Supplementary Material 1

## Data Availability

Data availability All data generated or analyzed during this study are included in this article.
