# Supplementary material for: Immobilization of silver nanoparticles and silver iodide within bamboo fabrics for wastewater treatment
Source: Sci Rep. 2025 Apr 1;15:11050. doi: 10.1038/s41598-025-93188-x (PMC11962099; doi:10.1038/s41598-025-93188-x)
Supplement: Supplementary file 1 — Supplementary Material 1 [file 41598_2025_93188_MOESM1_ESM.docx]

**Supplementary file**

**
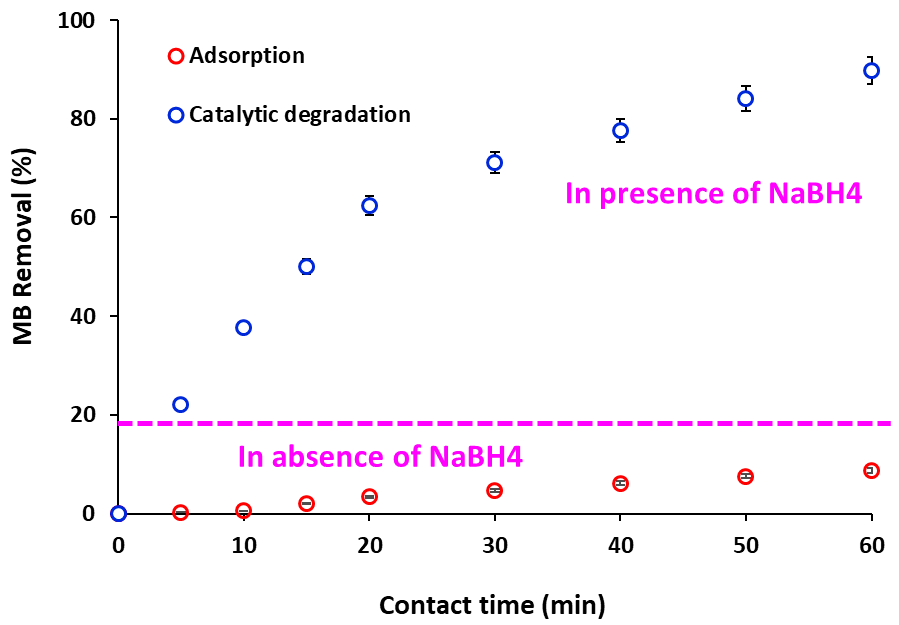
**

**Figure S1: The removal percentage of MB dye by AgNPs@AgI@Bamboo in absence (adsorption) and presence of NaBH_4_ (catalytic degradation).**


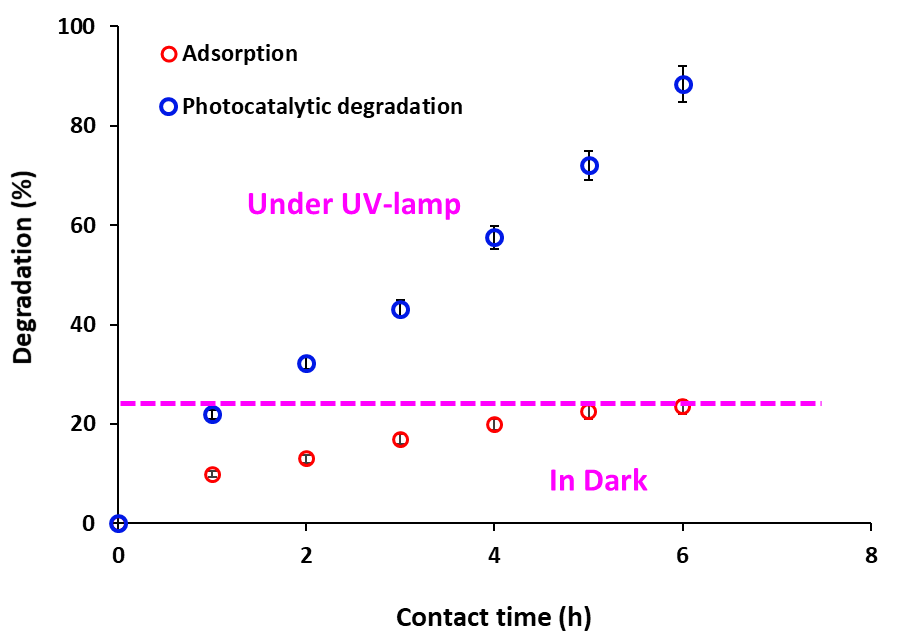


**Figure S2: The removal percentage of MB dye by AgNPs@AgI@Bamboo in absence (adsorption) and presence of UV light (photocatalytic degradation).**
